# Supplementary material for: Are carbon emissions trading and green financial instruments synergistic? -Comprehensive quantitative research based on content analysis
Source: PLoS One. 2024 Mar 7;19(3):e0298601. doi: 10.1371/journal.pone.0298601 (PMC10919601; doi:10.1371/journal.pone.0298601)
Supplement: S1 Data — (ZIP) [file pone.0298601.s001.zip › Data/地级市Z_F工作报告.zip!/░▓╗╒/╦▐╓▌╩╨/└┤╫╘╣1⁄2╓┌║┼í╛┬φ┐╦╩2╛▌═°í┐_2010.docx]

2010年1月19日宿州市第三届人民代表大会第二次会议

市长  吴旭军

各位代表：

现在，我代表市人民政府，向大会报告工作，请予审议，并请市政协委员和其他列席人员提出意见。

一、2009年工作回顾

2009年是我市经济社会在挑战中发展、在困难中前进的一年。面对国际金融危机的冲击，全市人民在中共宿州市委的坚强领导下，认真贯彻党的十七大和十七届三中、四中全会精神，紧紧围绕“双千亿、双翻番”的奋斗目标，全面实施三大战略，把保持经济平稳较快发展作为首要任务，全力做好保增长、保民生、保稳定的各项工作，圆满实现了市三届人大一次会议确定的主要目标。初步统计，全市生产总值完成560亿元，增长13%左右，连续四年保持两位数增长，三次产业结构调整到28.6：34.4：37。财政收入32.6亿元，增长18.6%；固定资产投资295亿元，增长40%以上；社会消费品零售总额168亿元，增长18.6%；城镇居民人均可支配收入13000元，农民人均纯收入4100元，分别增长10%、12%。

一年来，我们重点做了六个方面的工作：一是有效应对危机，保增长成效显著。针对金融危机不断蔓延的局面，我们见事早、行动快、措施准，出台一系列保增长的政策措施，促进了全市经济的企暖回升。一年中，我们始终把保企业作为保增长的重中之重，加大企业帮扶力度，提升要素保障能力。深入开展“四企”活动，优化企业发展环境。积极落实结构性减税政策，取消或减免涉企收费35个大项、70个子项。建立中小企业贷款风险补偿专项基金，注资1.6亿元成立中小企业投资担保公司，举办多场银企对接活动，全年发放各类贷款196亿元，为中小企业融资担保授信12.8亿元，发行企业债券8亿元，在关键时刻提振了企业信心。我们把扩投资作为保增长的主攻方向，抢抓国家扩大内需机遇，争取中央国债项目641个、资金6亿元，带动社会投资22亿元。深化项目提速行动和“项目建设年”活动，实行市四大班子负责同志联系重大项目制度，加强项目跟踪调度，钱营孜煤矿、京田机械、电光冶金、汇源果汁、南虹纺织二期等项目建成投产，一批输变电工程投入运行，京沪高铁、泗许高速、中元化肥、正鑫水泥等项目进展顺利，宿淮铁路、百丽鞋业、天瑞水泥等项目开工建设。我们把促消费作为保增长的切入点，实施家电、农机、汽车、摩托车下乡工程，农村消费增速八年来首次高于城市；及时出台促进房地产市场健康发展的政策，房地产业刚性需求加速释放，全市新开工面积354万平方米，竣工240万平方米。

二是扩张工业总量，产业结构不断优化。全面启动工业五年扩张计划，规模以上工业增加值完成150亿元，增长26%以上。规模以上工业企业增加到707家，其中亿元以上企业达到103家，新增22家。工业投资完成115亿元，增长36.5%。工业用电量增长10.5%，占全社会用电量的55%。工业经济综合效益指数达220%，创历史最好水平。与央企开展对接，签约项目总投资达184亿元。加快推进自主创新，新增省级企业技术中心3家、创新型企业1家、试点企业3家、省高新技术产品9项，载银活性碳纤维毡被列为国家级重点新产品，埇桥区荣获全国科技进步先进县（区）称号。出台加快非公有制经济发展的意见，建成各类创业基地18个，其中省级基地12个。大力推进名牌战略，梨苑牌精梳棉纱等6个产品获省名牌产品称号，新锦丰“味之家”被认定为中国驰名商标。强力开展环保风暴行动，集中整治各类污染源，强行关闭一批污染严重的小企业，奎河等重点流域污染治理工作初显成效，市、县污水处理管网建设加快，污染物总量减排和节能降耗目标基本完成。

三是强化农业基础地位，农村经济较快发展。全面落实各项强农惠农政策，发放各类涉农补贴9.7亿元。奋力夺取抗旱保苗的全面胜利，深入推进小麦高产攻关和玉米振兴计划，粮食连续六年丰收，总产达380万吨。组织实施“千区万场”规模化推进工程，畜牧业规模养殖比重提高6个百分点，泗县跻身全国生猪调出大县。大力实施农业产业化提升行动，规模以上农产品加工业产值达151亿元，增长14%，市级以上龙头企业完成营销收入178亿元，农民专业合作组织总数突破千家，萧县成为全国小麦加工业示范基地，泗县被命名为中国山芋之乡，埇桥区连续荣获全国产粮产油大县（区）称号。加快农田水利基本建设，治理易涝农田168万亩，完成岳庄坝水库建设及26座小水库除险加固，灵璧、萧县被列为全国小农水建设重点县。加强农村基础设施建设，农业综合开发稳步推进，全面完成广播电视“村村通”工程建设任务，建成农村公路“村村通”工程938公里，新增造林8.7万亩、农村沼气用户6042户，新建农村饮水安全工程138处，解决29万农村人口饮水安全问题。培训新型农民4.7万人，劳务输出稳定在140万人。深化农村综合改革，兽医管理体制改革、基层农技推广体系改革和村庄整治全面推进，林权登记发证任务基本完成。新农村建设“百村十镇”示范工程取得新进展。县域经济在结构调整中快速发展，对全市经济增长的贡献率达55%。乡镇经济不断壮大，财政收入过千万元乡镇达到12个。

四是加速城镇扩容，城乡建设稳步推进。相继完成中心城市总体规划修编评估报告、功能片区及职教园区建设规划编制。加大城市基础设施投入，“六路一桥一馆”全面建成，两大医院综合楼主体竣工，三角洲生态公园、家乐福世纪广场、华夏世贸广场进展顺利，新增绿地92万平方米，绿化覆盖率达36%，城市品位显著提升。集中开展城市综合整治，市容市貌进一步改观。加快老城区改造步伐，完成小街小巷改造21条。扩大低收入群体住房保障面，新建廉租房面积近19万平方米。各县县城建设步伐加快，基础设施建设逐步完善，新区开发进展顺利，副中心地位和功能不断提升。小城镇规划体系基本完成，集聚功能不断增强。与中国电信、中国移动签署战略合作框架协议，“数字宿州”与信息化建设加快推进。

五是深化改革开放，发展活力明显增强。深化部门预算、政府采购、国库集中支付和“收支两条线”制度改革，公共财政支出管理制度逐步完善。国有集体企业改制稳步推进。市政府机构改革、事业单位改革进展顺利，工商系统市场办管脱钩、国土资源基层站所改革任务全面完成。政府还贷二级公路收费站全部取消。加快地方金融体系建设，泗县农村合作银行成功组建，6家小额贷款公司获准筹建，农村信用社央行专项票据兑付工作全部完毕，我市被列为全省唯一的全国农村金融产品和服务方式创新试点市。深入实施招商突破行动，坚持领导带头招商，统筹推进产业招商、专业招商和集中招商，实际到位内资220亿元，增长40%，位居皖北六市第一；实际利用外资1.1亿美元，增长41%。成功举办第五届黄淮海地区农博会和第四届国际腐蚀控制大会，展示了宿州良好形象。与马鞍山市开展结对合作，达成多项合作意向。加快各类开发区建设，宿州经济开发区基础建设不断完善，县区开发区产业集聚能力进一步增强，28个新型乡村工业园区和工业集聚区初具规模。外贸进出口总额完成8400万美元。

六是坚持民生优先，社会建设步伐加快。大力实施28项民生工程，投入各类资金18.8亿元，惠及500多万城乡居民。新增城镇就业岗位2.6万个，城镇登记失业率控制在4.1%以内。扩大社会保险征缴面，实行市级集中征缴，五大险种参保人数增长5.3%。广泛开展爱国卫生运动，手足口病和甲型H1N1流感疫情得到有效防控。新型农村合作医疗参合农民达458.6万人，住院实际补偿比居全省第二。城市低保社会化发放实现全覆盖，农村低保标准提高到1000元，五保户集中供养率达到25%以上。继续推进义务教育均衡发展，免收81万中小学生义务教育阶段学费，免费提供中小学教科书资金1.1亿元，宿州二中新校区建成使用。组建了宿州技师学院。文化建设精彩纷呈，庆祝建市十周年系列文化活动和百戏进百乡深受好评，乡镇文化站、农家书屋等加快推进。竞技体育再创佳绩，在十一届全运会上获得金银牌各1枚。加快旅游资源开发和景区建设，组织开展百团万人游皖北和宿州人游宿州活动，石龙湖国家湿地公园获得正式命名。计生基层基础工作全面加强，出生人口性别比稳步下降，人口和计划生育综合考评在皖北地区保持领先。继续实行开门接访、带案下访，信访秩序进一步好转。安全生产监管、突发公共事件应急体系建设明显加强，质量和安全年活动扎实有效，食品药品放心工程深入实施。“五五”普法有序推进。组织开展严打整治百日会战、社会治安集中整治等专项斗争，顺利完成国庆60周年安保任务，人民群众安全感进一步增强。灵璧县被评为全国平安建设先进县。民族宗教和谐局面继续巩固，砀山县荣获全国民族团结进步模范集体称号。全力保障空降机动万人千车在宿集结演练，国防动员、人防和双拥工作取得新进步。残疾人、老年人和妇女儿童权益得到保护，人事人才、红十字、编制、审计、统计、物价、供销、扶贫开发、生产救灾、防震减灾、烟草、气象、档案、外宣、对台、地方志等工作均有新进展。

过去一年，是本届政府的开局之年。全市上下众志成城、共克时艰，经受住了金融危机的考验，各级政府的施政水平和领导能力，在应对挑战抓“三保”中得到进一步提升。围绕建设开放型、服务型、法治型和廉政型政府，进一步规范政府工作程序。自觉接受人大法律监督、政协民主监督和社会舆论监督，注意听取各方意见和建议，按期办结人大议案3件、代表建议126件、政协委员提案304件。深入开展学习实践科学发展观活动，巩固“四民”活动成果，组织“百个科室万人评”，促进了机关作风转变。进一步密切与工会、共青团、妇联等人民团体的联系。大力推进依法行政，全面推行政务公开。深化行政审批制度改革，进一步清理市级行政审批事项，取消19项，下放到县区33项，审批时限平均缩短11个工作日。扎实开展“小金库”治理工作，强化行政监察和审计监督，惩治和预防腐败体系建设不断加强。

各位代表，在宏观环境极其复杂的条件下，我们取得了超过预期的发展成绩，向新中国60华诞献上了一份厚礼。这主要得益于市委的坚强领导，得益于全市上下的奋力拼搏，得益于社会方方面面的关心支持。在此，我代表市人民政府，向全市广大干部群众，向各民主党派、工商联和人民团体，向驻宿解放军、武警官兵和政法干警，致以崇高的敬意！向关心、支持宿州发展的中央、省驻宿单位和海内外友好人士，向在我市创业的投资者、建设者，表示衷心的感谢！

各位代表，在看到成绩的同时，我们也清醒地认识到，尽管我市经济增速已连续四年稳定在两位数区间，但在省内人口总量靠前、经济总量居中、人均指标靠后的状况没有根本改变；尽管三次产业结构已实现历史性转折，但产业层次不高、过度依赖资源型产业的格局没有根本改变。同时，经济效益不高，发展后劲不足，财政保障能力不强等问题依然突出，一些地方和部门思想解放不够、发展氛围不浓、工作作风不实、办事效率不高等问题还不同程度存在。面对当前来之不易的良好发展态势，面对全市上下求富思变的强烈期盼，我们必须万众一心、开拓奋进，努力开创宿州经济社会发展新局面！

二、2010年工作总体要求

今年是全面完成“十一五”规划目标，衔接“十二五”发展的关键之年。综观大势，全球经济呈回升态势，我国继续处在发展的战略机遇期，中央保持宏观经济政策的连续性和稳定性，支持中西部地区发展的力度不断加大，导向更加鲜明，重点更加突出，措施更加具体，总体有利于我市发挥后发优势，抢抓经济回暖的发展机遇。就我市自身而言，经过多年积累，正跨入工业化、城镇化加速推进的新阶段，迈上持续较快发展的新平台。更为重要的是，经过金融危机的洗礼，各级干部应对复杂局面、谋划发展的能力更加成熟，干事创业、奋发图强的斗志更加旺盛，完全有条件把握新机遇、迎接新挑战、取得新成绩。但当前经济回升的基础还不稳固、不平衡，经济发展中的不稳定、不可预料因素还很多。我们必须准确把握当前宏观形势，进一步凝聚全市人民的力量与智慧，坚持科学发展不动摇，坚持加快发展不懈怠，坚持和谐发展不折腾，努力加速崛起步伐。

今年政府工作的总体要求是：以邓小平理论和“三个代表”重要思想为指导，深入贯彻落实科学发展观，紧紧围绕“双千亿、双翻番”的奋斗目标，以扩大投资、调整结构为主线，大力实施工业扩张、农业提升和城镇扩容战略，加快“两区两园”建设，加速壮大经济总量，积极培育新兴产业，加快推进改革开放，更加注重改善民生，促进社会稳定和谐，努力实现经济社会平稳较快发展。

主要目标是：全市生产总值增长13%以上；财政收入保持同步增长；规模以上工业增加值增长25%以上；固定资产投资和招商到位资金分别增长30%；工业固定资产投资增长35%；社会消费品零售总额增长18%；城乡居民收入均增长10%；人口出生率控制在13‰以内；城镇登记失业率控制在4.5%以内；单位生产总值能耗、化学需氧量和二氧化硫排放量达到省控目标。

在具体工作中，应着力把握以下几点：一要坚持好中求快。唯有保持较快发展速度，才能赢得发展先机，这是顺势而为的必然选择，是刻不容缓的历史使命。我们一定要顺应千帆竞发的时代大潮，呼应百舸争流的发展大势，快跑而不小跑，快进而不小进，尽快使经济实力大起来、综合竞争力强起来。二要善于趋利避害。政策因时而调、因势而变。我们一定要提高敏锐度、增强应变力，适时而动、因势利导，集众人之智，合众人之力，在结合上做文章，在创造上下功夫，力求少走弯路。三要务求干字当头。机遇稍纵即逝。今天的宿州，既等不得，更耽误不得，无论遇到多少艰难险阻，我们都要咬定青山不放松，按照认准的道路走下去，遵循既定的目标干下去，真抓实干，少说多干，埋头苦干，谱写发展的新篇章。四要谨记以人为本。突出发展的普惠性和公共服务的均等性，把经济增长的成效充分体现到民生改善上来，办成一批顺民心、合民意的好事实事，着力提高城乡居民生活水平，进一步凝聚力量，促进和谐。

三、促进经济持续稳定增长

立足发展第一要务，坚持增投入、调结构、扩内需、强基础，着力做好六项重点工作。

（一）千方百计加速经济回升。把扩大投资和刺激消费结合起来，把促进即期增长与长远发展结合起来，巩固当前来之不易的发展势头。

坚定不移扩大投资。把握国家投资导向，狠抓项目推进，继续以“三铁三高三路两河”和“五煤五电”为抓手，逐一制定推进方案，促进项目建设再有新突破。大力推进在建项目，力促百丽鞋业、雨润食品、天瑞水泥、戈尔管材、硕果电器建成投产，泗许高速一期、206国道改造项目早日通车，浍河蕲县船闸如期竣工，京沪高铁、宿淮铁路、泗许高速二期和徐明高速、市区到蒿沟高铁站连接线、元邦钢铁、汇源果汁二期、德利服饰和金黄庄、金狮、骑路孙煤矿加快建设，郑徐客运专线、国电宿州电厂、盛达铁矿、海螺水泥、萧县合成革和陶瓷产业基地尽早开工，以建设进度保障投资速度。加强城镇基础设施和城乡电网等项目建设，改善国省道干线及重要县道，建设500千伏埇桥输变电等电网工程。加强项目前期工作，力争使华电二期、张大屯和朱楼煤矿尽早落地，加快推进大唐煤电、皖能宿州电厂、皖北矸石电厂、济祁高速公路、新汴河综合治理工程等项目的前期工作。积极抢抓国家扩大内需的机遇，顺应产业政策，抓好项目对接和向上争取，加强基础设施、结构调整、节能环保、民生项目的谋划和储备，确保“6+3”工程储备规模超过2000亿元，省“861”项目储备规模超过1400亿元。完善领导联系重大项目制度，加强项目分类调度，提高项目服务水平和效率。认真落实各项推进非公有制经济发展的政策措施，放宽市场准入，降低创业门槛，落实税费优惠，激发全民创业的动力和活力。

提升消费对经济增长的拉动作用。顺应消费结构升级，积极扩大商贸、旅游、文化等消费规模，引进新型流通业态和新的流通方式，培育新的消费热点，促进节假日消费，主动引导消费预期，扩大即期消费规模。大力开拓农村消费市场，继续推进“万村千乡”市场工程，加大农村流通网络建设力度，提高“农家店”覆盖率。组织落实好家电、农机、汽车、摩托车下乡，启动汽车、家电以旧换新，加快优质耐用消费品进入农村市场的步伐。完善住房公积金制度，推进廉租住房、经济适用房等保障性住房建设，扩大普通商品房供给，促进房地产市场健康发展。

（二）推动工业结构优化升级。继续实施工业五年扩张计划，坚持一手抓总量扩张，一手抓结构调整，提升工业对经济增长的主动力。

做大做强主导产业。依托煤电能源、农副产品加工、建材、机械制造等产业基础，以骨干企业为依托，强化政策扶持，加快外延扩张，延伸产业链条，壮大规模实力，形成一批特色鲜明、体系完整的产业集群，加快打造千亿元产业基地。深化企业内涵改造，重点推进虹光6万吨胚芽油等30个投资3000万元以上的技改项目，支持企业提高装备水平，引导企业提升产业层次。认真落实相关扶持政策，加快企业退城进园建设步伐。支持皖北药业、安特生化等企业实施战略性重组，打造知名品牌，培育一批顶天立地的骨干企业，力争规模以上工业企业达到800家，其中过亿元企业110家，超10亿元企业10家。加大拟上市企业辅导、扶持力度，推动优势企业上市融资。全面落实促进中小企业发展的政策措施，加快建设政策服务、创业发展、人才培育、银企对接和科技创新平台。

加快培育战略性新兴产业。结合省十大新兴产业振兴规划纲要，组织专门班子，实施重点攻关，力争有更多项目列入省级规划。加大力度，推进企业自主创新，整合各类扶持资金，组织实施5个重大科技专项和重点科技项目，推动多种形式的产学研合作，形成一批具有核心竞争力的关键技术和优势产业。注重培养、引进和用好人才，建设具有创新创业能力的人才队伍。

强力推进节能减排。推进高碳产业低碳化改造和低碳技术应用，抓好中元化工等节能改造项目，加强40家重点耗能企业的节能监察。加大流域污染和重污染企业治理力度，加快污水处理管网建设，坚决完成省政府下达的节能减排任务。

（三）进一步加快城镇化步伐。按照中央积极稳妥推进城镇化的要求，坚持城乡统筹，同步推进中心城市、城镇与新农村建设。

加速中心城市发展。围绕中心城市“双百”目标，深入开展“城市建设管理年”活动。充分发挥埇桥区、宿州经济开发区的作用，共同建设皖东北中心城市。遵照南延、北扩、东进的思路，完成城市和土地利用总体规划，六个专项规划、五个重点区域和两个组团规划编制工作，以规划引领城市发展。坚持以项目为抓手，以大投入促进城市大发展，今年将组织实施“618”工程，计划投资60亿元，围绕道路畅通、环境提升、安居保障、公共公益、商贸流通、民生建设、文明创建和老城改造等8个方面，重点建设100个工程项目，进一步拉开城市框架，提升城市功能。围绕建设汴河新区政务文教中心、开发区和西南片区物流商贸新区、道东和沱东片区循环经济园区，分片制定推进计划，创新开发模式、管理方式、运行机制、投融资体制，推进城市组团发展。按照“降低密度、转移功能、缓解交通”的原则，加快中心城区和人民路两侧综合开发，改善老城区居住环境。加大对城郊结合部、城中村、棚户区的改造力度，继续推进小街小巷综合治理。围绕创建省级文明城市和国家级园林城市，健全城市管理长效机制，提升城市服务能力和服务水平。高标准推进广场绿化、道路绿化、庭院绿化，延伸城市绿化空间。

大力促进城乡统筹。完成砀城、灵城总体规划和泗城总体规划审批，完成乡镇总体规划修编调整和重点村庄建设规划编制。加快各县县城建设步伐，加大投入力度，完善路网建设，提升配套功能，推进新区开发，拉开县城框架。加快城乡基础设施体系、公共服务设施体系向农村的延伸，逐步形成布局合理、功能明确、结构完善的城镇体系。加快中心镇建设，有效吸纳和转移农村劳动力，增强辐射带动力。加快新农村建设，积极向上争取项目和资金，突出教育培训、医疗卫生、基础设施建设，继续办好“水电路气房”五件实事，改善农业生产和农民生活条件。统筹推进采煤塌陷区综合治理试点，努力使塌陷区农民原有生活水平不降低、长远生计有保障。

（四）持续加强“三农”工作。多渠道增加“三农”投入，促进农业增效、农民增收和农村稳定。

加快现代农业发展。以争创国家农村改革试验区和现代农业示范区为契机，全面推进现代农业建设。制定粮食、果蔬、畜牧和林木产业发展规划、现代农业八个支撑体系建设规划和现代农业核心区综合发展规划，出台配套政策，创新体制机制。继续实施农业产业化“532”提升行动，做大做强皖王等国家级龙头企业，培育壮大省级龙头企业，积极发展市级龙头企业，力争市级以上龙头企业达到200家，营销收入突破200亿元。充分发挥农民专业合作社作用，带动农民在农产品加工销售环节增加收入。优化种植业结构，巩固南粮、北果、中部经济作物的特色优势产业带，大力发展无公害、绿色和有机食品，建设一批安全优质农产品生产示范基地，培育一批名优品牌。实施好粮食增产规划，开展粮食高产创建活动，推进第五轮小麦高产攻关和玉米振兴计划。推进畜牧水产规模化健康养殖，加大支持力度，建设一批高标准养殖小区。继续实施好农田水利基本建设三年规划，抓好易涝农田和洼地综合治理，加强农田防护林建设，推进土地治理项目，优化农机装备结构，改善农业生产条件。全面落实各项强农惠农政策，认真执行小麦最低收购价，完善农业保险财政补贴。大力推广沼气、秸秆利用等可再生能源技术。强化农民负担监管和重点监控，严厉查处各种乱收费行为。

不断壮大县域经济实力。全面落实省委、省政府加快皖北和沿淮部分市县发展政策，进一步下放各类经济社会管理权限。引导县区立足自身资源禀赋，把握特色定位，选准适合本地条件、适应结构升级的产业，有针对性承接产业转移，构建有竞争力的产业体系，提升在全省县域经济综合排名中的位次。全面落实扩权强镇的政策措施，以规划为龙头、产业为支撑，加快经济结构调整步伐，因地制宜发展特色经济、板块经济，促进乡镇经济增速提质。认真落实发展乡村工业试点意见，加快推进乡村工业集聚区和农民工返乡创业园建设，突出抓好试点工作。

（五）深入推进改革开放。加快重点领域和关键环节改革，进一步扩大对外开放，增强发展活力。

推进体制机制改革创新。稳妥推进事业单位人事制度、收入分配制度改革。继续推进供销系统企业和粮食流通体制改革。在保持农村土地承包关系稳定的基础上，按照依法自愿有偿原则，鼓励农民以转包、转让、出租、互换、股份合作等多种形式流转农村土地承包经营权。大力开展农村土地整治，改善农村居住环境，实现土地集约经营，盘活农村建设用地资源。稳步推进城乡一体化综合配套改革，加快集体林权制度配套改革。巩固完善为民服务全程代理制，推进村级公益事业建设一事一议财政奖补试点、“惠民直达工程”试点。扎实推进基层医药卫生体制综合改革，创新体制机制，强化政府举办基层医疗卫生机构的公益性质，为群众提供优质、安全、价廉的医疗服务。继续深化国有集体企业改革，增强企业发展活力。健全综合治税机制，加大清费治乱力度，继续落实好结构性减税政策，调整优化支出结构，积极培植财源，更好地发挥财政对转变发展方式、保障和改善民生的支持作用。

持之以恒推进招商引资。主动参与泛长三角发展分工协作，积极举办各类主题招商活动，逐步增强招商引资的针对性、有效性。主攻重点企业、重点行业特别是500强企业，大力招引战略合作伙伴和配套企业，切实引进一批有助产业结构优化升级的项目。完善招商体制，改善招商条件。坚持领导带头招商、小分队驻点招商，深化产业招商、专业招商、以商招商、企业招商、行业商会招商，拓宽引资渠道。抓好在手项目的跟踪落实，提高招商项目履约率、资金到位率和项目开工率。继续强化与央企的对接，深化与马鞍山市的合作，在更广领域、更深层次取得实实在在的进展。办好第四届灵璧石文化节，积极参加上海世博会，广交商界朋友，展示宿州形象。积极扩大进出口贸易，落实出口促进和鼓励政策，抓好浓缩果汁、板材、服装等出口基地建设，着力培植一批市场竞争力强的出口龙头企业。

全力加快开发区建设。按照3至5年全市六个省级开发区面积超百平方公里、产值过千亿的目标，进一步完善、提升市县（区）开发区发展规划，加快基础设施建设，增强承载能力，提高投资强度，把开发区打造成集聚产业、集聚资本、集聚人气的平台。围绕争创国家级开发区，加快宿州经济开发区建设，打造产业、资金、物流三大链条，规划建设五大产业园，努力构筑以工业经济为主体、以现代物流园和商住新城区为两翼的发展格局，当好全市经济发展的龙头。

（六）培育壮大现代服务业。把发展现代服务业作为经济结构调整的重要着力点，加快建设皖东北商贸中心。

拓展服务业发展内涵。着力打造中心商圈，加快大型商贸服务设施建设，加快物流园区规划建设，加快各类综合市场和专业市场建设，推动商业地产开发，发展连锁经营、电子商务等现代流通方式，进一步优化市场结构和网点布局，全面提升现代商务贸服务功能。特别要依托高速公路、铁路建设，争取国家对黄淮海地区小麦流出基地建设的支持，引进省内外知名商贸企业和物流企业，打造煤炭、粮食、钢材、农资等一批现代物流基地。制定完善旅游业发展总体规划和各专项规划，注重文化内涵提升，加快闵祠、虞姬和钟馗文化园建设，推进蔡洼红色旅游，深度开发五柳、皇藏峪、黄河故道风景区，高水准打造一批旅游精品，尽快提升旅游业发展水平。积极发展中介、金融保险、信息等新型现代服务业态，大力发展融资、评估、担保等中介服务组织。紧紧围绕扩大内需、改善民生的需要，加快教育、卫生、交通、通信等方面配套功能建设，发展社区服务、教育培训、健身休闲、养老保健等服务业。

提高金融创新能力和服务水平。积极适应适度宽松货币政策的变化趋势，加强政府对金融工作的指导。改善和强化政银企合作，落实与金融机构签署的战略合作框架协议。加快金融产品和服务方式创新，发挥好国有商业银行、农村信用社的主渠道作用，加大信贷投放力度，缓解中小企业融资困难。继续争取交通银行、马鞍山农村商业银行在我市设立分支机构，支持徽商银行加快县域网点布局，引进金融机构在泗县、灵璧设立村镇银行，加快农村信用社向农村合作银行过渡，推进灵璧农村合作银行正式挂牌，逐步形成多元化、多层次的地方金融体系。加快组建小额贷款公司、农民资金互助合作社等新型农村金融组织，解决农村发展资金难题。加强银行业监管，引导民间金融机构规范发展，推进金融生态县区和农村信用体系建设，改善金融生态环境。

四、推动经济社会协调发展

坚持以人为本，办好利民之事，尽好安民之责，让人民群众共享更多的改革发展成果。

精心实施民生工程。加大财政支出向民生领域的倾斜力度，集中力量为人民群众多办实事。按照省政府统一部署，将民生工程调整扩充到33项，新增中小学校舍安全工程、社会（儿童）福利中心、农村留守儿童之家、光荣院建设和农村清洁工程5项，扩面提标农村居民最低生活保障、城乡卫生服务体系建设、新型农村合作医疗、城镇居民基本医疗保险、政策性农业保险、中等职业学校农村困难学生和涉农专业学生免费6项内容，惠及更多城乡居民。建立稳定多元的筹资机制，及时足额落实配套资金，抓好工程类项目建设。强化督促检查，健全绩效考核评价管理制度，确保民生工程扎实推进。

完善就业和社会保障体系。突出高校毕业生、农民工和就业困难群体，加强技能培训、政策帮扶和援助，鼓励支持更多的劳动者自主创业。认真落实减轻企业负担稳定就业岗位的各项政策，组织开展多种形式的就业援助活动，完善创业培训、小额贷款、创业孵化等服务体系，多渠道拓展就业岗位。提高企业退休人员养老金。建立低保标准自然增长机制，提高五保对象的供养水平，提高困难群体、优抚群体、孤老孤残孤儿等特殊群体的生活保障水平。稳步扩大社会保险覆盖面，加快基本医疗保险、工伤保险市级统筹。积极推进新型农村养老保险，逐步实现被征地农民社会保险制度全覆盖。

协调发展各项社会事业。推进义务教育均衡发展，合理配置教育资源，全面推进素质教育，继续优化中小学布局，加快农村寄宿制学校建设，提高农村留守儿童和进城农民工子女教育水平。加强教师队伍建设，实施义务教育学校教师绩效管理。提升高中阶段教育水平，一中新校区力争明年秋季招生。加强职业教育，加快职教园区建设，促进宿州技师学院新体制尽快运转，支持宿州卫校升格。巩固完善新型农村合作医疗制度，提高筹资标准，参合农民补助标准提高到每人每年120元，参合率达到92%以上。加强医疗机构监管，坚决控制医疗费用不合理增长，让参合群众得到更多实惠。继续加强基层医疗卫生服务体系建设，开工建设开发区医院，完成县医院、中医院、乡镇卫生院、村卫生室建设工程任务。稳妥推进公立医院改革，支持三级医院创建工作。继续抓好甲型H1N1流感等传染病防治，严防疫情大范围传播。推进公共卫生均等化服务，提高公共卫生服务水平。充分挖掘整合文化资源，推进文化精品创作，广泛开展特色群众文化活动，大力发展文化事业和文化产业，重视文化基础设施建设，加快建设文化产业园，构建公共文化服务体系。传承弘扬宿州精神，加强公民思想道德教育，加快市民活动中心建设，不断提高市民素质。认真贯彻落实全民健身条例，积极备战省十二运会。完善流动人口管理服务体系，综合治理出生人口性别比，千方百计稳定低生育水平。认真做好第六次全国人口普查工作。全面贯彻落实党的民族政策和宗教工作基本方针，依法管理宗教事务，推动清真寺和清真食品供应网点加快建设。支持工会、共青团、妇联等人民团体参与经济社会管理和公共服务。推进档案、地方志、外事、侨务、对台、地震、气象、老龄、红十字工作，促进残疾人事业发展。

维护社会和谐安定。严格落实安全生产的政府监管责任和企业主体责任，加大重点领域、特殊行业、关键部位隐患排查治理力度，开展交通、防火安全等专项整治，严防重特大事故发生。畅通民意表达渠道，认真解决群众的合理诉求，做好新形势下各类群体性事件和其他突发事件规律性研究，健全预测预警机制和应急预案体系，把影响稳定的问题解决在基层、解决在萌芽状态。加强法制宣传教育，认真开展普法和依法治理工作。改善政法系统基础条件，推进公安“三项”建设，重视农村社会治安综合治理，依法严厉打击各种违法犯罪活动，打造平安宿州。大力整顿和规范市场秩序，加强食品药品安全集中整治和日常监管。加强价格监测和预警，确保重要商品市场价格稳定。支持驻宿部队建设，加强国防动员、民兵预备役和人防工作，再创全国双拥模范城。

各位代表，宿州的发展正处在奋力起跳的重要关口，各级政府要按照“四型”机关的要求，全面加强自身建设，推动经济社会在科学发展轨道上健康运行。一要加强学习实践，增强发展本领。各级政府及其工作人员，要按照学习型机关的要求，创新学习方法，健全学习制度，进一步巩固扩大学习实践活动成果，在保增长、调结构、惠民生的实践中，着力提高战略思维、创新思维、辩证思维能力，着力提高谋划发展、推动发展的水平，不断增强服务群众、维护稳定的本领。二要强化宗旨意识，提高服务能力。坚持以人为本、人民唯上，强化为企业、为基层、为社会的服务理念，时刻关注企业经营状况，倍加关心群众冷暖疾苦，切实解决基层困难，全力保持社会安定。巩固深化“四民”、“四企”活动成果，不断提高政府决策科学化、民主化水平。加快推进政务公开，促进政府与企业、市民交流互动，问政于民、问需于民、问计于民，提高政府工作透明度和满意度。三要立足高效务实，提升行政效能。全面推进依法行政，继续深化行政审批制度改革，推行“两集中、两到位”，推进基建项目联合审批大平台建设，加大县区行政服务中心建设力度，优化经济发展环境。切实精简会议、压缩文件，减少事务性活动，把更多的时间和精力放在解决问题上，放在工作落实上。加大督查和行政问责力度，确保政令畅通。自觉接受人大法律监督和政协民主监督，认真听取各民主党派、工商联、无党派人士及人民团体的意见和建议，重视做好人大代表议案、建议和政协委员提案办理工作。四要坚持从严治政，倡树清廉正气。坚持“一岗双责”，严格执行廉洁从政各项规定，加快推进惩治和预防腐败体系建设。加强重大项目监察审计，认真执行和不断完善项目招投标、土地出让、政府采购等制度，深入推进工程建设领域突出问题专项治理，从源头上预防腐败发生。牢固树立过紧日子的思想，自觉与广大人民群众同甘共苦。保持清廉本色，自觉做到思想不松防线，行动不碰红线，做人不越底线，努力营造风清气正的社会环境。

各位代表！新的形势下，把宿州崛起大业持续推向前进，是我们光荣而艰巨的历史任务。让我们高举中国特色社会主义伟大旗帜，更加紧密地团结在以胡锦涛同志为总书记的党中央周围，在市委的坚强领导下，同心同德，艰苦奋斗，合力攻坚，为全面开创宿州加速崛起的新局面，为建设开放、繁荣、和谐的新宿州而努力奋斗！
